# Antibacterial Activity of the Essential Oil of *Piper tuberculatum* Jacq. Fruits against Multidrug-Resistant Strains: Inhibition of Efflux Pumps and β-Lactamase

**DOI:** 10.3390/plants12122377

**Published:** 2023-06-19

**Authors:** Lucas Yure Santos da Silva, Cicera Laura Roque Paulo, Talysson Felismino Moura, Daniel Sampaio Alves, Renata Torres Pessoa, Isaac Moura Araújo, Cícera Datiane de Morais Oliveira-Tintino, Saulo Relison Tintino, Carla de Fatima Alves Nonato, José Galberto Martins da Costa, Jaime Ribeiro-Filho, Henrique Douglas Melo Coutinho, Grażyna Kowalska, Przemysław Mitura, Marek Bar, Radosław Kowalski, Irwin Rose Alencar de Menezes

**Affiliations:** 1 Laboratory of Pharmacology and Molecular Chemistry, Department of Biological Chemistry, Regional University of Cariri-URCA, Crato 63105-000, CE, Brazil; lucas.yure@urca.br (L.Y.S.d.S.); trabalho.renata18@gmail.com (R.T.P.); 2 Laboratory of Microbiology and Molecular Biology, Department of Biological Chemistry, Regional University of Cariri-URCA, Crato 63105-000, CE, Brazil; lauraroquealencar@gmail.com (C.L.R.P.); talysson.f.moura@urca.br (T.F.M.); daniel.sampaio10@urca.br (D.S.A.); isaac.moura@urca.br (I.M.A.); datianemorais@gmail.com (C.D.d.M.O.-T.); saulorelison@gmail.com (S.R.T.); hdmcoutinho@urca.br (H.D.M.C.); 3 Laboratory Natural Products Research, Department of Biological Chemistry, Regional University of Cariri-URCA, Crato 63105-000, CE, Brazil; carlaalvesbio@hotmail.com (C.d.F.A.N.); galberto.martins@gmail.com (J.G.M.d.C.); 4 Oswaldo Cruz Foundation (FIOCRUZ), Fiocruz Ceará, R. São José, S/N-Precabura, Eusébio 21040-900, CE, Brazil; jaime.ribeiro@fiocruz.br; 5 Department of Tourism and Recreation, University of Life Sciences in Lublin, 15 Akademicka Str., 20-950 Lublin, Poland; grazyna.kowalska@up.lublin.pl; 6 Department of Urology and Oncological Urology, Medical University of Lublin, 8 Jaczewskiego Str., 20-954 Lublin, Poland; przemyslaw.mitura@umlub.pl (P.M.); barmarek1@gmail.com (M.B.); 7 Department of Analysis and Food Quality Assessment, University of Life Sciences in Lublin, 8 Skromna Str., 20-704 Lublin, Poland

**Keywords:** *Piper tuberculatum*, essential oils, multidrug-resistant bacteria, phytochemical analysis

## Abstract

Antimicrobial resistance has become a growing public health concern in recent decades, demanding a search for new effective treatments. Therefore, this study aimed to elucidate the phytochemical composition and evaluate the antibacterial activity of the essential oil obtained from the fruits of *Piper tuberculatum* Jacq. (EOPT) against strains carrying different mechanisms of antibiotic resistance. Phytochemical analysis was performed using gas chromatography–mass spectrometry (GC/MS). The antibacterial activity of EOPT and its ability to inhibit antibiotic resistance was evaluated through the broth microdilution method. The GC-MS analysis identified 99.59% of the constituents, with β-pinene (31.51%), α-pinene (28.38%), and β-cis-ocimene (20.22%) being identified as major constituents. The minimum inhibitory concentration (MIC) of EOPT was determined to assess its antibacterial activity against multidrug-resistant strains of *Staphylococcus aureus* (IS-58, 1199B, K2068, and K4100). The compound showed a MIC of ≥ 1024 μg/mL, suggesting a lack of intrinsic antibacterial activity. However, when the EOPT was associated with antibiotics and EtBr, a significant decrease in antibiotic resistance was observed, indicating the modulation of efflux pump activity. This evidence was corroborated with the observation of increased fluorescent light emission by the bacterial strains, indicating the involvement of the NorA and MepA efflux pumps. Additionally, the significant potentiation of ampicillin activity against the *S. aureus* strain K4414 suggests the β-lactamase inhibitory activity of EOPT. These results suggest that the essential oil from *P. tuberculatum* fruits has antibiotic-enhancing properties, with a mechanism involving the inhibition of efflux pumps and β-lactamase in MDR *S. aureus* strains. These findings provide new perspectives on the potential use of EOPT against antibiotic resistance and highlight the importance of *Piper* species as sources of bioactive compounds with promising therapeutic activities against MDR bacteria. Nevertheless, further preclinical (in vivo) studies remain necessary to confirm these in vitro-observed results.

## 1. Introduction

*Staphylococcus aureus* is the leading cause of endocarditis, bacteremia, osteomyelitis, and infections of the skin and soft tissues [1]. *S. aureus* strains are of great clinical interest, especially in the nosocomial environment, as they can colonize healthy individuals asymptomatically [2], contributing to their spreading. In addition, the increase in antibiotic resistance rates indicates that the prevalence of *S. aureus*-associated diseases will continue to increase [3].

With the acquisition of resistance mechanisms, micro-organisms can evade microbial control methods, resulting in ineffective management, persistence, and spread of infections [4]. Evidence has indicated that the inappropriate and indiscriminate use of antibiotics leads to the selection of resistant strains favoring their dissemination, representing a significant challenge for global health policies. In this context, it is estimated that in 2050, diseases caused by bacterial infections will be responsible for the deaths of millions of people and will cost public and private coffers trillions [5].

Efflux pumps are transmembrane proteins conserved in many living organisms, including bacterial and human cells. It has been consistently demonstrated that these proteins decrease the intracellular accumulation of drugs, thus reducing their efficacy. Thus, efflux pump expression is one of the most unique antibiotic resistance mechanisms [6]. Another mechanism that considerably impacts antibiotic resistance is the expression of β-lactamases, enzymes that catalyze the hydrolysis of β-lactam antibiotics [7].

Therefore, searching for effective and safe inhibitors of efflux pumps and β-lactamases is a promising strategy to combat antibiotic resistance [8]. The possibility of using efflux pump inhibitors to restore the activity of antibiotics has long been addressed experimentally, showing promising results in the development of therapeutic adjuvants [9], as these inhibitors allow the reuse of antibiotics that have already been shown to be safe and effective for clinical use [10].

Due to advances in chemistry and the isolation of secondary metabolites, producing semisynthetic and synthetic compounds has been considered an advantage for studying and developing new drugs [11]. Naturally derived compounds serve not only as new drugs but also as therapeutic adjuvants capable of improving the effectiveness of existing drugs [12].

Medicinal plants are widely used by traditional communities in Brazilian folk medicine. Notably, in the Northeast Region, various species known as caatinga plants have had their pharmacological potential demonstrated through many studies [13]. Medicinal plants have great importance in therapeutics. According to the World Health Organization (WHO), 85% of the therapeutic products used in traditional medicine are plants and their products, including extracts and active principles [14].

Species of the genus *Piper* have been traditionally used to treat venereal diseases, intestinal disorders, genitourinary ailments, and epilepsy, as well as to prevent conception. The literature has shown that several fixed constituents are found in species of this genus and, therefore, can play a role in their biological effects [15]. *Piper tuberculatum* Jacq., popularly known as monkey pepper, belongs to the Piperaceae family and has notable economic and medicinal value. This species can be found in many regions of Brazil, where it is used empirically as a sedative antidote for snake venom and to treat stomach diseases [16]. Previous research has demonstrated the biological activities of *P. tuberculatum*, revealing its antimicrobial, insecticidal, and antiprotozoal effects [17,18,19].

Essential oils consist of a complex mixture of terpenes with diverse functional groups, occurring in different concentrations and demonstrating a great diversity of biological activities and mechanisms of antibacterial action, including the inhibition of target enzymes, induction of cell membrane rupture, and inhibition of protein synthesis [20]. However, the relationship between *P. tuberculatum* essential oil composition and its effects on antibiotic resistance remains to be investigated.

Therefore, this study aimed to determine the phytochemical composition and characterize the antibacterial activity of the essential oil of the fruits of *P*. *tuberculatum* Jacq. against multidrug-resistant strains of *S*. *aureus* expressing efflux pumps (NorA, Tet(K), and MepA) and β-lactamase.

## 2. Results

### 2.1. GC-MS Profile of Piper tuberculatum Essential Oil

The GC-MS analysis of *Piper tuberculatum* essential oil identified 99.59% of the constituents, including β-pinene (31.51%), α-pinene (28.38%), and β-cis-ocimene (20.22%) as the major compounds, as shown in Table 1.

### 2.2. Antibacterial Activity and Antibiotic Modulation in Efflux Pump-Carrying S. aureus Strains

**Figure 1:**
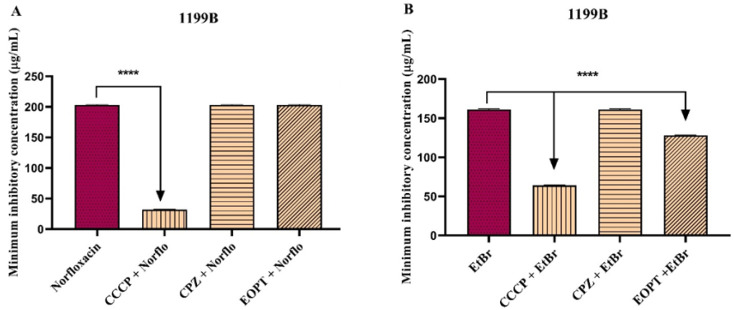
Effect of the essential oil of *Piper tuberculatum* fruits (EOPT) in combination with norfloxacin (**A**) and ethidium bromide (**B**) against *S. aureus* strain 1199B, which expresses the NorA efflux pump. **** = *p* < 0.0001 vs. control was determined using one-way ANOVA followed by Dunnett’s post hoc. Norflo = norfloxacin; CPZ = chlorpromazine; EtBr = ethidium bromide.

The results showed that EOPT had no direct antibacterial activity against *S. aureus* strains 1199B, IS-58, and K2068 since its MIC was ≥1024 μg/mL (not shown). However, when combined with antibiotics and EtBr, EOPT exhibited different modulation profiles. In tests with strain 1199B, the EOPT combination had no impact on the norfloxacin MIC (Figure 1A). However, the essential association significantly reduced the MIC of EtBr compared to that of the control, suggesting that EOPT can interfere with the activity of the NorA protein (Figure 1B).

**Figure 2:**
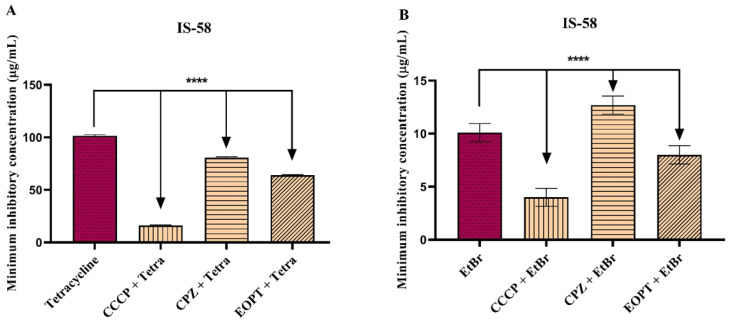
Effect of the essential oil of *Piper tuberculatum* fruits (EOPT) in combination with tetracycline (**A**) and ethidium bromide (**B**) against *S. aureus* strain IS-58, which expresses the Tet(K) efflux pump. **** = *p* < 0.0001 vs. control was determined using One-way ANOVA followed by Dunnett’s post hoc. Tetra = tetracycline; CCCP = carbonyl cyanide 3-chlorophenylhydrazone; CPZ = chlorpromazine; EtBr = ethidium bromide.

When the EOPT was combined with the antibiotic tetracycline or EtBr against the IS-58 strain, a significant reduction in the MIC of both compounds was observed, suggesting a potentiation of antibiotic resistance associated with the inhibition of the Tet(K) efflux pump (Figure 2A,B).

**Figure 3:**
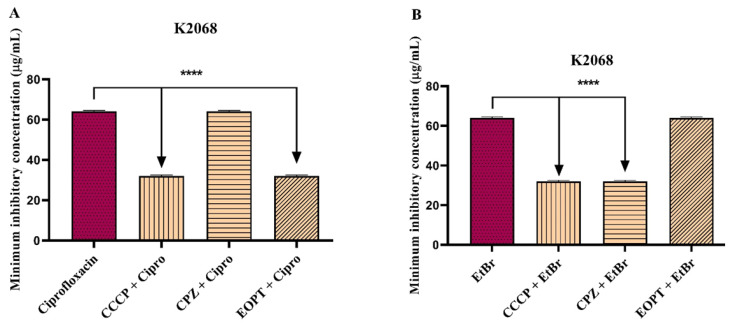
Effect of the essential oil of *Piper tuberculatum* fruits (EOPT) in combination with ciprofloxacin (**A**) and ethidium bromide (**B**) against *S. aureus* strain K2068, which expresses the MepA efflux pump. **** = *p* < 0.0001 vs. control was determined using One-way ANOVA followed by Dunnett’s post hoc. Tetra = tetracycline; CCCP = carbonyl cyanide 3-chlorophenylhydrazone; CPZ = chlorpromazine; EtBr = ethidium bromide.

In tests with strain K2068, the association with EOPT also reduced the MIC of ciprofloxacin and EtBr, suggesting potential antibiotic potentiation involving the inhibition of the MepA efflux pump (Figure 3A,B).

### 2.3. Evaluation of NorA and MepA Efflux Pump Inhibition by Fluorescence Emission

**Figure 4:**
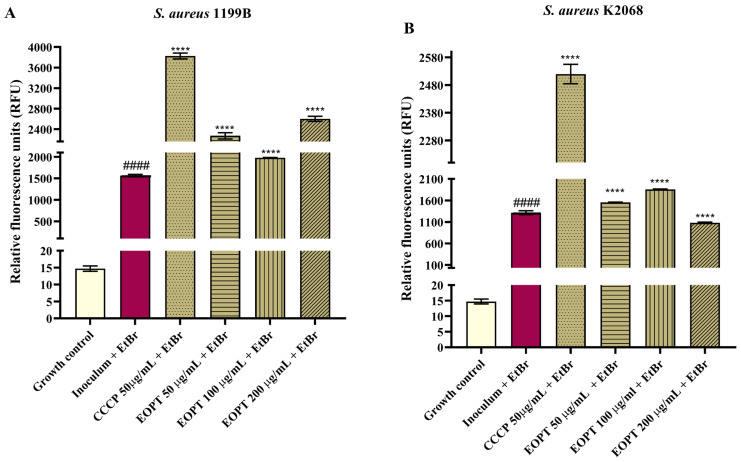
Evaluation of NorA and MepA inhibition by *Piper tuberculatum* essential oil in an EtBr fluorescence emission assay. (**A**) *S. Aureus* 1199B (NorA). (**B**) *S. Aureus* K2068 MepA. #### = *p* < 0.0001 vs. growth control and **** = *p* < 0.0001 vs Inoculum + EtBr.

A fluorescence emission assay addressed the evidence that the EOPT can inhibit *S. aureus* efflux pumps. It was observed that fluorescence emission in *S. aureus* strain 1199B was increased following the exposition to EOPT 50, 100, and 200 μg/mL compared to the negative control (inoculum + EtBr) (Figure 4A). Significant inhibition was also observed in the pharmacological control group treated with the standard inhibitor CCCP. In *S. aureus* strain K2068, EOPT at 50, 100, and 200 μg/mL was also able to increase the fluorescence emission of EtBr compared to the control (Figure 4B).

**Figure 5:**
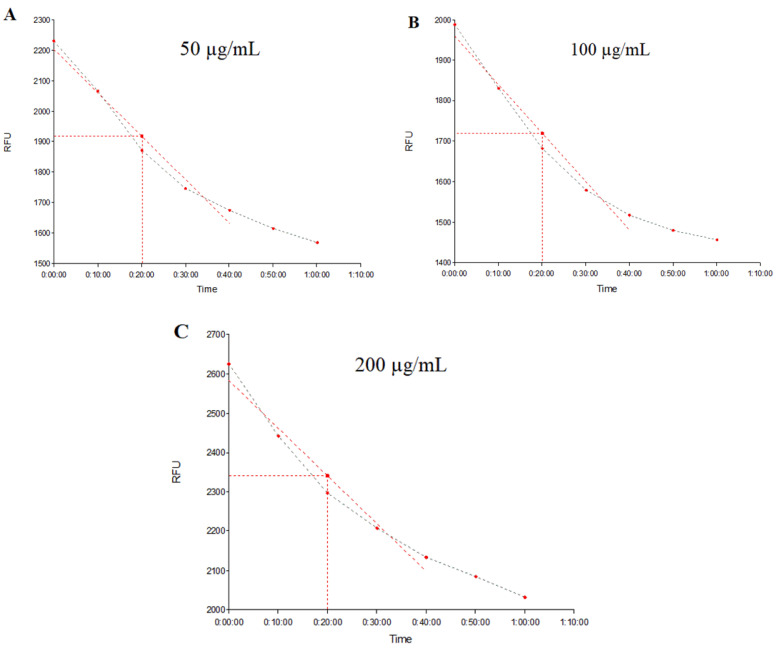
Evaluation of fluorescence emission by the *S. aureus* 1199B strain over time. (**A**–**C**) represent groups treated with essential oil of *Piper tuberculatum* at 50, 100, and 200 μg/mL, respectively.

**Figure 6:**
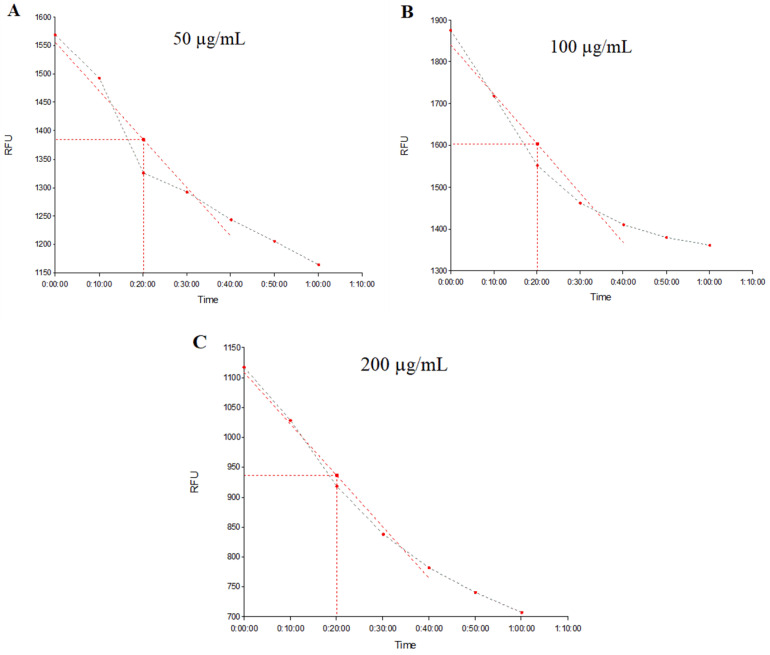
Evaluation of fluorescence emission by the *S. aureus* K2068 strain over time. (**A**–**C**) Represent groups treated with the essential oil of *Piper tuberculatum* at 50, 100, and 200 μg/mL, respectively.

Figure 5 demonstrates the real-time (at 10 min, 20 min, 30 min, 40 min, 50 min, 1 h, and 1 h 10 min) fluorescence emission in strain *S. aureus* 1199B treated with EOPT at 50 μg/mL, 100 μg/mL, and 200 μg/mL (A, B, and C, respectively), while Figure 6 shows the fluorescence emission rate by strain K2068 treated and evaluated under the same conditions. The decreasing curve over time indicates the decrease in fluorescence emission due to EtBr efflux through the functional efflux pump.

### 2.4. Evaluation of β-Lactamase Inhibition

**Figure 7:**
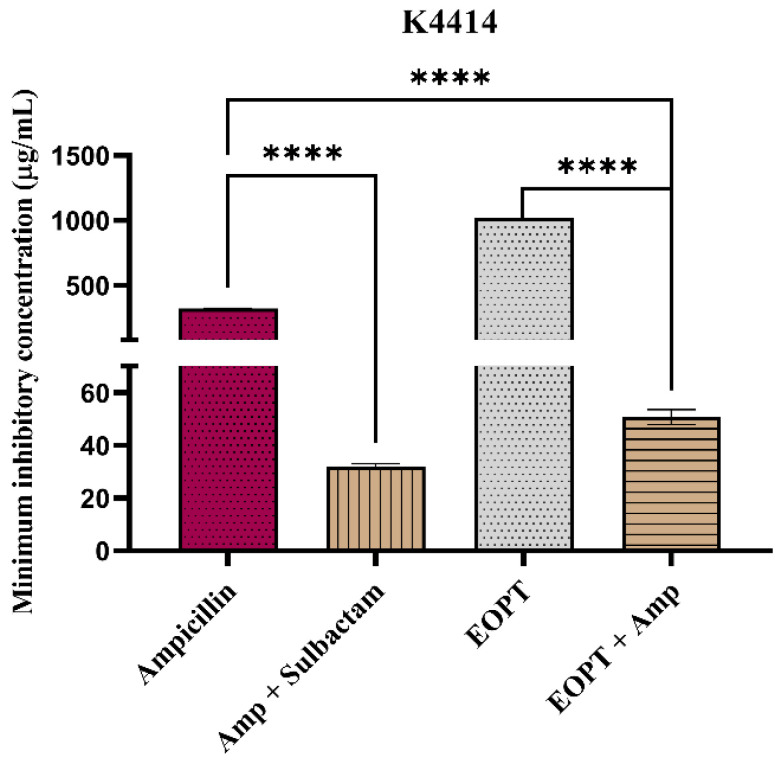
Evaluation of β-lactamase inhibition through antibiotic MIC reduction in *S. aureus* strain K4414 cultures treated with the essential oil of *Piper tuberculatum* fruits (EOPT). One-way ANOVA followed by Dunnett’s post hoc test. Amp = ampicillin; **** = *p* < 0.0001 vs. control.

The K4414 strain of *S. aureus* is known to present β-lactamase-mediated resistance to antibiotics and is used to evaluate potential β-lactamase inhibitors [21,22]. It was observed that the combination with the essential oil reduced the MIC of ampicillin by more than three-fold, indicating an increase in the antibacterial effect of ampicillin associated with the EOPT, possibly due to β-lactamase inhibition. The standard inhibitor sulbactam caused comparable inhibition of the ampicillin MIC, corroborating this hypothesis (Figure 7).

## 3. Discussion

*Piper* species produce a variety of volatile compounds, such as monoterpene hydrocarbons (e.g., α-pinene), oxygenated monoterpenoids (e.g., linalool), sesquiterpene hydrocarbons (e.g., β-caryophyllene), and oxygenated sesquiterpenoids (e.g., caryophyllene), the occurrence of which may vary according to seasonality, geographic region, genotype, and environmental factors [23]. Thin et al. [24] proposed a classification of this genus based on the dominant class of compounds present in essential oils (EOs), i.e., monoterpenes (e.g., *Piper demeraranum* and *Piper tuberculatum*), sesquiterpenes (e.g., *Piper nigrum*), monoterpenes and sesquiterpenes (e.g., *Piper aduncum*), phenylpropanoids (e.g., *Piper marginatum*), benzenoid compounds (e.g., *Piper harmandii*) and non-terpenoid compounds (e.g., *Piper maclurei*).

Comparing the present results of the chemical characterization with those presented in the work of Santos Sales et al. (2018), studying an essential oil obtained from the same species, it was possible to observe the presence of the same major constituents: β-pinene and α-pinene [25]. These constituents were also identified in studies investigating other species in the genus, albeit at lower concentrations [26,27,28].

Although the mode of antibacterial action of terpenes remains largely unknown, studies have shown that most terpenoids can inhibit vital microbial processes, such as oxygen uptake and oxidative phosphorylation [29]. The monoterpenes α-pinene and β-pinene, found in essential oils of many plants, have a wide range of reported pharmacological activities, among which antibacterial activity stands out, being associated with their toxic effects on cell membranes [30]. β-ocimene, also identified as one of the major constituents of the oil under study, is a monoterpene capable of performing various biological functions in plants. It was demonstrated that this compound could affect floral visitors and mediate defensive responses to herbivory [31].

Species of *Piper* are reported as natural sources of phytochemical compounds that can be helpful in the treatment of infections and various other diseases. In the literature, many studies report the antimicrobial activity of essential oils obtained from species belonging to this genus [32,33,34,35]. These promising results are attributed to the bioactive compounds that the essential oil is composed of, considering that the determining factors of the bioactivity of essential oils are their composition, the functional groups present in the active components, and their synergistic interactions [36].

Previous research investigating extracts and oils obtained from *Piper tuberculatum demonstrated* its antimicrobial action against bacterial and fungal strains [37,38,39,40]. Studies with essential oils obtained from other species of *Piper* also found significant activity against the strains analyzed in this study [41,42,43,44]. In tests conducted with the oil of *Piper tuberculatum* fruits, Santos Sales et al. (2017) found a MIC of ≥1024 μg/mL against the strains used in the study, corroborating the present research findings. Chouhan, Sharma, and Guleria (2017) reported that bioactive components present in essential oils can adhere to the surface and cellular structures and subsequently penetrate the cell membrane, impairing cell metabolism and leading to cell lysis [45].

The irrational use of antimicrobials significantly contributed to the selection of multidrug-resistant (MDR) pathogens, impairing the treatment of many infectious diseases, principally those caused by bacterial strains. Evidence indicates that the efflux pump expression, structural alteration of the target, and enzymatic degradation of drugs are effective mechanisms of resistance that should be considered in antibacterial drug development [46,47,48,49,50]. In this context, secondary metabolites of plants can increase the effectiveness of conventionally used antibiotics [51] by promoting synergistic interactions that potentiate the effect of clinically used clinical antibiotics such as norfloxacin, which has become ineffective due to the increase in antimicrobial resistance [52,53].

Investigating the action of β-pinene and α-pinene on the growth of bacterial strains, Leite et al. (2007) observed significant antibacterial effects [54]. In addition, De Araújo et al. (2021) identified α-pinene, one of the significant constituents of EOPT, as a possible inhibitor of the NorA efflux pump [55]. This compound decreased the MICs of EtBr and norfloxacin. It showed favorable interaction with this protein in a molecular docking analysis, suggesting that α-pinene may be partially responsible for the effects observed in this study [29].

Norfloxacin, tetracycline, and ciprofloxacin are substrates for the efflux pumps NorA, Tet(K), and MepA, respectively, while EtBr is an unspecific efflux pump substrate. It has experimentally been observed that the reduction in MIC efflux substrates such as antibiotics and EtBr indicates efflux pump inhibition by a test compound. Therefore, it is reasonable to assume that OEPT contains components that act as inhibitors of NorA, Tet(K), and MepA in *S. aureus* strains [56,57]. Additionally, the increased fluorescence emission by the strains indicates that the essential oil possibly inhibited the NorA and MepA efflux pumps. EtBr is a DNA-intercalating agent emitting more fluorescence the more it is bound to the genetic material. Therefore, efflux pump inhibition increases the intracellular concentration of EtBr [58,59,60,61].

Freitas et al. (2021) also investigated α-pinene as a possible TetK and MrsA efflux pump inhibitor [62]. They found that this monoterpene has promising effects against efflux pump-carrying *S. aureus* strains, reducing the MIC of EtBr and the antibiotics required, indicating that this compound may help combat antibacterial resistance. This finding also points to the involvement of α-pinene in the effects observed in the present study.

To date, there are no records in the literature regarding the use of the compounds α-pinene, β-pinene, and β-ocimene as β-lactamase inhibitors. However, evidence indicates that terpenes have excellent antimicrobial activity against antibiotic-resistant strains. In this context, terpinolene showed promising antibiotic-enhancing effects due to the inhibition of efflux pumps and β-lactamase [63,64].

With the therapeutic use of safe and effective β-lactam antibiotics, the prevalence and variety of β-lactamases have multiplied dramatically over the past decades [65]. β-lactamases are bacterial enzymes that hydrolyze the β-lactam bonds in antibiotics, rendering them non-functional [66]. Alongside improvements in β-lactam antibiotics themselves, combinations of susceptible β-lactams with mechanism-based β-lactamase inhibitors represent the primary strategy to combat β-lactamase-mediated resistance, and natural products and their derivatives have shown promise as inhibitors [67].

## 4. Materials and Methods

### 4.1. Plant Material and Extraction of the Essential Oil

The fruits of *P. tuberculatum* Jacq. were collected in the municipality of Crato, State of Ceará, Brazil. The samples were collected in triplicate, dried, and taken to the herbarium. Dr. Maria Arlene Pessoa da Silva performed the botanical identification. Then, the voucher specimen was deposited in the Herbarium Caririense Dárdano de Andrade Lima (HCDAL) of the Regional University of Cariri (URCA) cataloged under registration number 24.2022.

The fresh fruits (6546 g) were subjected to hydrodistillation in a Clevenger-type apparatus. The material was weighed and placed in a glass flask to which distilled water was added and boiled for 2 h. At the end of this period, the extracted essential oil was treated with anhydrous sodium sulfate to eliminate residual moisture and stored in an amber glass bottle.

### 4.2. Determination of the Chemical Profile of the Piper tuberculatum Essential oil via CG-MS

The essential oil was analyzed using a Shimadzu GC–MS QP2010 series, provided by Shimadzu Scientific Instruments Inc. (Columbia, MD, USA), with fused a silica capillary column, SH-Rtx-5 (30 m × 0.25 mm I.D.; 0.25 m film thickness), and the following temperature program: 80–180 °C at 4 °C/min, then 246 °C at 6.6 °C/min, closing with 10 min at 280 °C, at 3.4 °C/min, totaling to an analysis time of 30 min. Helium was used as the carrier gas, with a 1.5 mL/min flow rate, split mode (1:100), and the injection port set at 220 °C. The quadrupole MS operating parameters were the following: interface temperature (280 °C) and ion source (200 °C); electron impact ionization at 70 eV; scan mass range of 40–350 *m*/*z* with a sampling rate of 1.0 scan/s; injection volume of 1 μL of a 500 ppm solution prepared with dichloromethane. The constituents were identified through a computational search using digital libraries of mass spectral data (NIST 08) and by comparing their authentic mass spectra and those reported in the literature [68].

### 4.3. Bacterial Strains and Culture Media

The strains used in this study were the following: *S. aureus* 1199B (which carries the NorA protein and is resistant to hydrophilic fluoroquinolones); *S. aureus* IS-58 (which carries the Tet(K) protein and is resistant to tetracyclines); *S. aureus* K2068 (which carries the MepA efflux pump); and *S. aureus* K4100 (which expresses the β-lactamase enzyme). All strains were maintained in culture media with specific antibiotics at subinhibitory concentrations to ensure that each strain’s resistance gene was expressed. Heart Infusion Agar (HIA, Difco laboratories Ltd., São Paulo, Brazil), prepared and diluted following the manufacturer’s recommendations, and 10% Brain Heart Infusion Broth (BHI Acumedia Manufacturers Inc., Baltimore, MD, USA) were used as culture media.

### 4.4. Substances

The antibiotics norfloxacin, tetracycline, ciprofloxacin, and ampicillin + sulbactam were used according to their specificity for each tested strain. The essential oil from the fruits of *Piper tuberculatum* (EOPT) and the antibiotics were dissolved in dimethyl sulfoxide (DMSO) and then dissolved in sterile distilled water. Ethidium bromide (EtBr), a DNA-intercalating agent used as a standard efflux pump substrate, was dissolved in sterile distilled water. Carbonylcyanide m-chlorophenylhydrazone (CCCP) was dissolved in a mixture of methanol:water (1:1, *v*/*v*), and chlorpromazine (CPZ) was diluted in DMSO and sterile distilled water. Both compounds were used as positive controls in the efflux pump inhibition tests. All substances were diluted to 1024 μg/mL, stored at 20 °C, and protected from light.

### 4.5. Minimum Inhibitory Concentration (MIC) Determination

The antibacterial activity of EOPT was verified by determining the minimum inhibitory concentration (MIC) through the broth microdilution method proposed by the authors of [69] with some adaptations. Bacterial cultures were grown in HIA medium 24 h before the experiments. On the day of the experiment, bacterial inocula were prepared in sterile saline solution according to the 0.5 value on the Mc Farland scale, corresponding to 1.5 × 10^8^ colony-forming units. Subsequently, solutions containing 900 μL of liquid BHI culture medium and 100 μL of the bacterial inoculum were prepared in test tubes. A volume of 100 μL of this solution was dispensed into each well in 96-well microtiter plates. Serial microdilution (1:1) was then performed with EOPT or antibiotics to reach concentrations ranging from 512 μg/mL to 0.5 μg/mL. The plates were incubated in the bacteriological oven at 37 °C for 24 h. For the readings, 20 μL of resazurin (7-hydroxy-3H-phenoxazin-3-one 10-oxide) was added to each well, and the color change of the medium was observed and interpreted as follows: red/pink indicated bacterial growth, while blue indicated growth inhibition. The MIC was the lowest concentration with no bacterial growth [70].

### 4.6. Evaluation of Efflux Pump Inhibition via Modulation of the MIC of the Antibiotics and EtBr

The MIC of antibiotics and EtBr was determined following the addition of EOPT to analyze the potential interference with efflux pump mechanisms. Test solutions containing BHI medium and 10% of the bacterial inoculum were prepared following the addition of EOPT at a subinhibitory concentration (MIC/8). Control solutions were prepared using only BHI medium and the bacterial inoculum. Then, 100 μL of these solutions were distributed in the wells on a 96-well plate. Serial dilutions (1:1) were performed with norfloxacin, tetracycline, ciprofloxacin, or EtBr to achieve drug concentrations ranging from 512 μg/mL to 0.5 μg/mL. After 24 h, the readings were performed as described above. Efflux pump inhibition was indicated by the reduction in MIC on plates containing EOPT associated with antibiotics or EtBr, compared to that of the negative control [71,72].

### 4.7. Analysis of NorA and MepA Inhibition by Increased EtBr Fluorescence Emission

*S. aureus* strains 1199B and K2068 were used and seeded on Heart Infusion Agar (HIA) and kept in a bacteriological oven at 37 °C 24 h before the experiments. As previously described, the inoculum was prepared in phosphate-buffered saline (PBS) in test tubes following the McFarland scale. Test tubes were filled with a solution containing the inoculum and EOPT at 50 μg/mL, EOPT at 100 μg/mL, or EOPT at 200 μg/mL. CCCP (50 μg/mL) was used as a positive control. The volume of the tubes (1 mL) was completed with PBS after adding the inoculum and the substances. The solutions were incubated, and then EtBr (100 μg/mL) was added, except in the growth control. The solutions were incubated again and, after 1 h, centrifuged at 10,000 rpm for 2 min and washed twice, followed by centrifugation. The readings were performed with the BioTek^®^ Cytation 1 fluorescence microplate reader and Gen5™ Software v. 3.0 using 530 nm excitation and a 590 nm emission wavelength. The readings were performed every 10 min from 0 to 1 h 10 min after washing. The groups included the following: growth control; negative control (inoculum + EtBr); positive control (inoculum + EtBr or CCCP); and test group (inoculum + EtBr and EOPT at 50 μg/mL, 100 μg/mL, or EOPT 200 μg/mL) adapted from Blair; Piddock, 2016b and Pal et al., 2020. [58,73]

### 4.8. Evaluation of β-Lactamase Inhibition

The *S. aureus* K4414 strain, which expresses the β-lactamase gene, was used for this test. The broth microdilution method was used to perform the assays, as described in item 2.5. In the negative control group, the action of ampicillin alone against K4414 was evaluated and compared to that of the ampicillin + EOPT group. In the positive control group, ampicillin was associated with sulbactam, a β-lactamase inhibitor. β-lactamase inhibition was considered when a three-fold decrease in the MIC of ampicillin was observed following the association of this antibiotic with EOPT [70].

### 4.9. Statistical Analysis

Data were analyzed via a one-way ANOVA and Bonferroni’s post hoc test using GraphPad Prism 5.0 software. All assays were performed in triplicate.

## 5. Conclusions

The present findings indicate that the essential oil obtained from the fruits of *P. tuberculatum* inhibits the antibiotic resistance mediated by β-lactamase and the efflux pumps NorA, Tet(K), and MepA in *S. aureus* strains. The antibiotic-enhancing potential attributed to EOPT may be related to the presence of terpenes, such as α-pinene, β-pinene, and β-ocimene. These are bioactive substances with broadly described antimicrobial activities, among which the potentiation of the activity of antibiotics directly links with the present findings. Nevertheless, further preclinical (in vivo) studies remain necessary to confirm these in vitro-observed results.

## Figures and Tables

**Table 1:** Chemical composition of the essential oil from *Piper tuberculatum* fruits.

Compounds		%	RT	IR ^1^	IR ^2^
1	α-pinene	28.38	3.99	1072	1071
2	Camphene	0.23	4.15	1092	1090
3	β-pinene	31.51	4.47	1117	1118
4	α-Phellandrene	0.23	4.75	1137	1140
5	β-terpinyl acetate	6.52	5.03	1157	1168
6	β-*cis*-ocimene	20.22	5.17	1166	1173
7	*cis*-sabinene hydrate	0.26	5.39	1182	1191
8	Linalool	0.63	5.65	1200	1202
9	Terpinen-4-ol	0.39	6.46	1242	1231
10	α-Terpineol	0.77	6.55	1246	1257
11	α-Copaene	1.06	8.26	1350	1351
12	*trans*-Caryophyllene	6.11	8.67	1381	1392
13	α-Humulene	0.41	8.98	1408	1413
14	Alloaromadendrene	0.18	9.06	1417	1423
15	α-Amorphene	0.14	9.14	1426	1437
16	Germacrene-D	1.08	9.23	1438	1444
17	byciclogermancrene	0.82	9.40	1457	1464
18	δ-Cadinene	0.14	9.59	1480	1494
19	Nerolidol	0.25	9.88	1511	1514
20	α-Muurolol	0.15	10.27	1545	1561
21	Caryophyllene oxide	0.11	10.40	1556	1565
TOTAL		99.59			
Monoterpenes		89.14			
Oxygenated monoterpenes		8.57			
Sesquiterpenes		10.45			
Oxygenated sesquiterpenes		0.51			

RT: Retention time; IR ^1^: obtained linear retention index; IR ^2^: linear retention index of the literature.

## Data Availability

The data presented in this study are available in the article.
